# Giant Hip Synovial Cyst Causing Deep Vein Thrombosis and Femoral Head Osteonecrosis in a Rheumatoid Arthritis Patient

**DOI:** 10.31138/mjr.33.3.328

**Published:** 2022-09-30

**Authors:** Vasiliki Syrmou, Antonios A. Koutalos, Maria Karapli, Ioannis Alexiou, Dimitrios P. Bogdanos, Christina G. Katsiari, Theodora Simopoulou

**Affiliations:** 1 Department of Rheumatology and Clinical Immunology, Faculty of Medicine, University General Hospital of Larissa, University of Thessaly, Larissa, Greece,; 2 Department of Orthopaedic Surgery & Musculoskeletal Trauma, Faculty of Medicine, University of Thessaly, Larissa, Greece

**Keywords:** synovial cyst, rheumatoid arthritis, osteonecrosis, deep vein thrombosis, hip

## Abstract

Hip synovial cysts are rare. However, in patients with Rheumatoid Arthritis (RA) they present in higher frequency than in general population. Herein, we present an unusual case of a 67-year-old man with RA that presented with unilateral leg oedema and Deep Vein Thrombosis (DVT). Computed tomography (CT) scan revealed a giant cystic lesion adjacent to the right hip joint with longitudinal diameter of 14 cm. Magnetic Resonance Imaging (MRI) confirmed the characteristics of the cyst. Interestingly enough, there was evidence of osteonecrosis of the femoral head. CT guided Fine Needle aspiration (FNA) of the fluid revealed fluid consistency similar to synovial fluid, while it excluded infectious process and malignancy. Patient was finally treated with total hip arthroplasty 3 months after the initiation of low molecular weight heparin (LMWH) in treatment dose.

## INTRODUCTION

Traditionally, cystic lesions related to hip joint are thought to be rare clinical entities. Cystic lesions can be either benign or malignant. Benign cystic lesions are further subdivided in synovial cysts, ganglion cysts and bursae.^1^ Synovial cysts differ from ganglion cysts in the presence of a lining of synovial cells surrounding the myxomatous fluid collection (synovial fluid). Synovial cysts arise after penetration of the synovium in the adjacent soft tissues either because of increased intraarticular pressure or as the result of a developmental defect.^2^ Synovial cysts are common in joints such as the knee, ankle, hand, and wrist, whereas the hip joint is a rare site of synovial cyst formation. Hip synovial cysts are mostly associated with degenerative joint conditions. In most cases, patients are asymptomatic and cysts are diagnosed incidentally on imaging studies. When enlarged, patients complain about groin or thigh pain, reduced range of movement, gait difficulty and manifestations due to compression of surrounding structures like vein stasis, vascular compromise, bladder and bowel disturbances, and nerve paralysis. In this case we describe the rare case of an RA patient presenting with a giant hip synovial cyst which caused leg oedema due to DVT and at the same time osteonecrosis of the femoral head. The diagnostic and therapeutic challenges are also discussed.

## CASE REPORT

**Figure 1.**
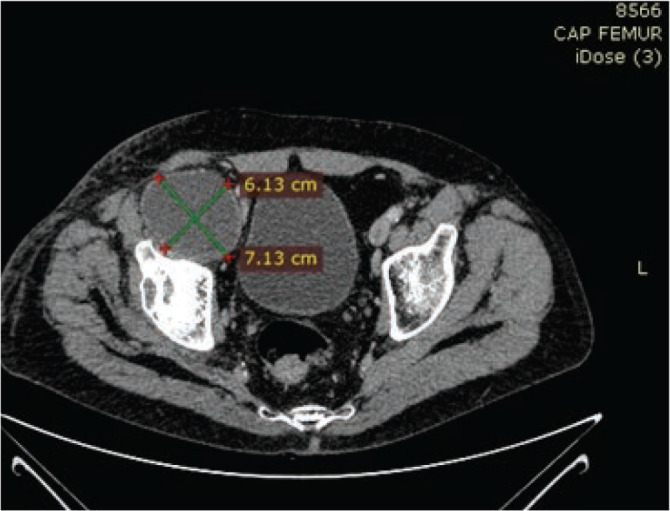
CT scan with IV contrast showing cystic lesion with contrast enhancement of its thin wall in conjunction with the right hip joint in front of the iliopsoas muscle.

**Figure 2.**
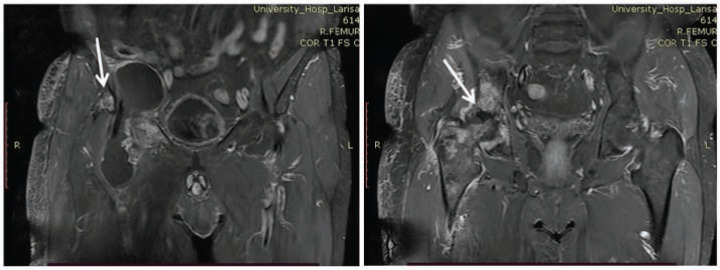
(**a**) MRI T1-weighted sequence with fat suppression showing the giant cystic lesion with 14,5 cm coronal dimension. There is excessive inflammation in the soft tissues surrounding the hip joint. (**b**) T1-weighted sequence with fat suppression showing osteonecrosis of the femoral head with complete destruction of its architecture.

A 67-year-old man with past medical history of seropositive RA (Rheumatoid Factor [RF] positive and anti-cyclic citrullinated peptide [a-CCP] negative) presented in the Emergency Department reporting painless unilateral right leg oedema. The patient was receiving leflunomide and adalimumab as treatment for RA. He denied injury of the limb and fever, while he noticed the oedema of the right leg getting progressively worse over the last two weeks. From physical examination, pulse was identified in femoral, popliteal, posterior tibial, and dorsalis pedis artery. Sensation was intact while there was restriction in the range of motion of the right hip joint (mainly in flexion and abduction) due to the oedema. There was circumference difference greater than 5 cm both at the mid-femur and mid-tibia level. Well’s score for DVT was 3 indicating high risk. As far as his blood results were concerned, C-Reactive Protein was found mildly increased (CRP= 1,76 mg/dl with upper normal limit= 0,5 mg/dl). Thrombosis in the proximal part of the superficial femoral vein was detected with right lower limb venous triplex. At the same time, in the right groin a large cystic lesion with 9 cm diameter was identified. The patient was started on treatment dose low molecular weight heparin (tinzaparin) and was admitted to the hospital. DAS28 score was 3.44, indicating moderate disease activity. CT scan of the thorax and abdomen with intravenous contrast was performed to further characterise the cystic lesion and to rule out malignancy and possible compression of nerves and large vessels of the pelvis. No expansion of the thrombus was seen in the large veins of the pelvis. The cystic lesion in the right groin was containing inhomogeneous fluid, had internal septa, as well as contrast enhancement of its thin wall. It was located in front of the right femoral head and the right iliopsoas muscle in conjunction with the right hip joint with axial level diameter 7x6 cm and coronal dimension of 14,5 cm. There was no evidence of compression of the large vessels of the pelvis (**Figure 1**). In the differential diagnosis groin abscess, lymph node block with central liquefaction, synovial cyst with inflammation and/or possible bleeding, epidermal cyst, and lymphangioma were included. MRI confirmed the characteristics of the lesion. However, a new manifestation has appeared, in the form of necrosis of the right femoral head with inflammation of periarticular soft tissues (fat and iliopsoas muscle tendon sheath) surrounding the hip joint (**Figure 2A and 2B**). Regarding the aetiology of osteonecrosis, no trauma was reported, and subsequent laboratory workup excluded the presence of antiphospholipid antibodies, hypertriglyceridemia, or hyperlipidaemia. The patient claimed no smoking or alcohol use and was not receiving corticosteroids.

**Figure 3.**
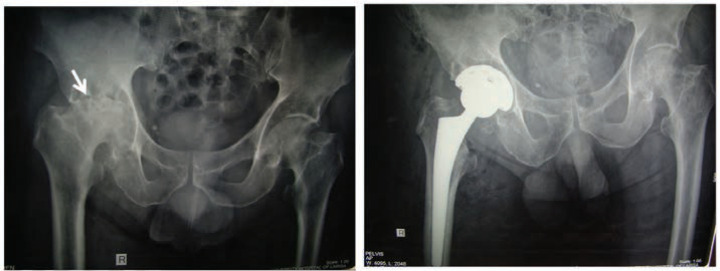
(**a**) Plain hip X-ray with complete destruction in the hip joint, significant bone degeneration and collapse of the femoral head in the acetabulum (before surgery). (**b**) hip joint after right total hip arthroplasty.

Given the increased risk for perioperative bleeding due to tinzaparin therapeutic dose and the increased risk for expansion of the thrombosis and/or pulmonary embolism decision was made for CT guided FNA of the cystic lesion. The microscopic examination of the aspirated fluid revealed 5800 WBC with 96% neutrophils, while the Gram stain was negative. No organisms and no mycobacteria were grown from the fluid culture, while in the fluid cytology no malignant cells were identified. The consistency of the fluid was similar to synovial fluid and the lesion was characterised as synovial cyst. After 3 months of anti-thrombotic treatment, right leg triplex was repeated, and complete resolution of the thrombus was confirmed, while the x-ray showed that the femoral head was totally destructed (**Figure 3A**). Tinzaparin was stopped and the patient underwent total hip arthroplasty (**Figure 3B**). Leg oedema resolved rapidly with spontaneous improvement in the range of movement.

## DISCUSSION

In general, hip synovial cysts are believed to comprise rare clinical entity. In literature, a few cases of large cysts have been described, causing significant symptomatology like inguinal masses, paraesthesia, nerve palsy and venous outflow obstruction and DVT. What makes this case of special interest, is the presence of a giant hip synovial cyst which caused both DVT and at the same time femoral head osteonecrosis. To our knowledge, there are only two published cases of huge hip synovial cysts leading to femoral head osteonecrosis.^3,4^ Avascular necrosis of the right femoral head was a rather unexpected finding, as the patient did not complain of any hip pain. From a pathophysiological point of view, osteonecrosis involves compromised subchondral microcirculation. Decreased femoral head blood flow can occur mainly through three mechanisms: vascular interruption due to fractures or dislocation, intravascular occlusion, or extravascular compression.^5^ Thus, most common risk factors include trauma, hypercoagulable syndromes, as well as alcohol and corticosteroid use that lead to an increase in extra-vascular pressure, hypothesized to create an obstruction in vascular flow leading to subsequent ischemia of the femoral head^5,6^ (**Table 1**). We made the assumption that the increased intraarticular fluid pressure led to critical reduction of femoral head perfusion and subsequent osteonecrosis.^7^ As far as the cause of the synovial cyst is concerned, RA seems to be the ultimate culprit. Excessive fluid accumulation in the synovium of the hip due to chronic inflammation probably strangulated the vulnerable perfusing vessel.

**Table 1.** Etiologic factors associated with femoral head osteonecrosis.

**Traumatic-associated risk factors**
Femoral neck fracture, femoral head fracture, dislocation of the hip joint
**Atraumatic-associated risk factors**
Corticosteroid use
Excessive alcohol intake
Coagulopathy (antiphospholipid syndrome, systemic lupus erythematosus, myeloproliferative disorders)
Other (Caisson disease-dysbarism, sickle cell disease, hemoglobinopathies, Gaucher disease, hyperlipidemia, hypertriglyceridemia, radiation therapy, chronic renal failure, chronic pancreatitis)

Osteonecrosis, as identified in pathology specimens of the femoral head, is found in 12% of RA patients undergoing hip arthroplasty and it occurs mainly in 2 forms, ie, classic avascular necrosis and degenerative osteoarthritis. Both types are correlated to glucocorticoid use.^8^ A recent large retrospective study on the prevalence of avascular necrosis among patients with prior oral corticosteroid therapy, found that low cumulative doses pose a small risk of development osteonecrosis.^9^ The development of osteonecrosis seems to be directly related to duration and total dosage of the medication.^10^ After reviewing our patient’s medical records, he had no need for glucocorticoids over the last five years and his RA seemed to be quite well controlled as indicated by medical records from outpatient’s clinic and inflammatory markers in laboratory results.

Ye at al. published a case series with 15 patients with femoral vein compression secondary to hip synovial cyst. In two of them there was DVT (13.3%) while all of them presented with unilateral leg oedema.^11^ As stated by Angelini et al degenerative joint diseases like osteoarthritis, rheumatoid arthritis and trauma are amongst the most common causes of synovial hip cysts.^12^ Other less common causes are developmental aplasia, labral tear, rapidly destructive arthrosis of the hip, juvenile idiopathic arthritis, polymyalgia rheumatica and hip arthroplasty.^1^

Multiple treatment options are available for synovial cyst of the hip joint, depending mainly on the severity of the symptoms due to local compression, which depend on the size of the cyst and its location. Needle aspiration, with or without injection of steroids or sclerosing agents may be an effective treatment in asymptomatic patients without vessel or nerve compression. Femoral and/or iliac vein compression, arterial compromise and nerve compression constitute emergencies and thus, they require early recognition by the clinician and immediate treatment. In the presence of thrombosis appropriate treatment should be initiated either by LMWH or even with vascular filter insertion. As far as the treatment of the cyst is concerned, total hip arthroplasty has been chosen in most cases in literature.^11,13^ However, aspiration and drainage under ultrasound or CT guidance remains an option despite the higher chances of regression.^11^ It has previously been reported that if the primary disease is RA, effective treatment may reduce the size of the cyst.^14^ DAS28 in this gentleman was not high but it became obvious that it did not reflect the level of degeneration of the right hip joint caused by RA. As the synovial cyst of the hip joint caused vein compression and was also complicated with osteonecrosis of the femoral head, total hip arthroplasty was the only therapeutic option. In this case, the basic challenge was the decision regarding the best time to operate. In the literature most of the large synovial cyst cases associated with significant symptomatology were treated surgically immediately. For this patient, perioperative bleeding risk due to treatment dose LMWH precluded a prompt surgical intervention. Performing radiologically guided aspiration of the cyst allowed to establish the diagnosis and most importantly excluded the possibility of malignancy and infection, thus giving adequate time to the patient to complete a full 3-month course of therapeutic dose tinzaparin.

In conclusion, we report a relatively rare case of a giant synovial cyst in a rheumatoid hip, a potential life-threatening complication, managed successfully with surgical excision and total hip replacement due to femoral head osteonecrosis.

## ABBREVIATIONS

Anti-CCP: Anti-cyclic citrullinated peptide antibodies
CT: Computed Tomography
DVT: Deep Venous Thrombosis
FNA: Fine Needle Aspiration
LMWH: Low Molecular Weight Heparin
MRI: Magnetic Resonance Imaging
RA: Rheumatoid Arthritis
RF: Rheumatoid Factor
